# Correlation between immune-related adverse events and prognosis in patients with various cancers treated with anti PD-1 antibody

**DOI:** 10.1186/s12885-020-07142-3

**Published:** 2020-07-14

**Authors:** Hiroshi Matsuoka, Takahiro Hayashi, Karen Takigami, Kazuyoshi Imaizumi, Ryoichi Shiroki, Naoki Ohmiya, Kazumitsu Sugiura, Kenji Kawada, Akira Sawaki, Koutaro Maeda, Yousuke Ando, Ichiro Uyama

**Affiliations:** 1 Department of Surgery Fujita Health University, Dengakugakubo 1-98, Kutsukake-cho, Toyoake City, Aichi Japan; 2 College of Pharmacy, Kinjo Gakuin University, 2-1723 Oomori Moriyama, Nagoya City, Aichi 463-8521 Japan; 3 Department of Respiratory Medicine Fujita Health University, Dengakugakubo 1-98, Kutsukake-cho, Toyoake City, Aichi Japan; 4 Department of Urology Fujita Health University, Dengakugakubo 1-98, Kutsukake-cho, Toyoake City, Aichi Japan; 5 Department of Gastroenterology Fujita Health University, Dengakugakubo 1-98, Kutsukake-cho, Toyoake City, Aichi Japan; 6 Department of Dermatology Fujita Health University, Dengakugakubo 1-98, Kutsukake-cho, Toyoake City, Aichi Japan; 7 Department of Clinical Oncology Fujita Health University, Dengakugakubo 1-98, Kutsukake-cho, Toyoake City, Aichi Japan; 8 Fujita Health University International Medical Center, Dengakugakubo 1-98, Kutsukake-cho, Toyoake City, Aichi Japan; 9 Department of Pharmacy Fujita Health University, Dengakugakubo 1-98, Kutsukake-cho, Toyoake City, Aichi Japan

**Keywords:** Immune-related adverse events, Programmed cell death-1, Nivolumab, Pembrolizumab

## Abstract

**Background:**

Immune checkpoint inhibitors (ICIs) targeting programmed cell death protein 1 (PD-1) are used for the treatment of various cancer types. However, immune-related adverse events (irAEs) occur in patients treated with ICIs. Several small-scale studies have reported the onset of irAEs and therapeutic effects of ICIs. Here we report a large-scale retrospective study covering a wide range of cancers. We evaluated irAEs and the therapeutic effects of ICIs and determined whether irAEs could be predicted.

**Methods:**

This study included patients treated with the anti-PD-1 antibodies nivolumab or pembrolizumab at Fujita Health University Hospital between December 2015 and March 2019. We retrospectively reviewed the electronic medical records for age, cancer type, pre-treatment blood test data, presence or absence of irAE onset, type and severity of irAEs, outcome of irAE treatment, response rate, progression-free survival and overall survival.

**Results:**

Two hundred-eighty patients received ICIs. The overall incidence of irAEs was 41.1% (115 patients), and the incidence of severe irAEs of grade 3 and higher was 2.8% (eight patients). The most common irAEs were skin disorders, thyroid disorders and interstitial pneumonitis. Patients with irAEs were significantly older than those without irAEs (69.7 versus 66.0 years, *P* = 0.02). The objective response rate (ORR) in patients with irAEs was 30.4%, which was significantly higher than in patients without irAEs (12.7%; *P* < 0.01). Both the median overall and progression-free survival were significantly longer in patients with irAEs (*P* < 0.01, *p* < 0.01). Based on the blood test data obtained before ICI therapy, hypothyroidism, thyroid-stimulating hormone levels and thyroglobulin antibody levels were associated with the onset of irAEs. In many patients with irAEs of Common Terminology Criteria for Adverse Events Grade 3 or higher, re-administration of ICIs was difficult, and their outcomes were poor. In contrast, many patients with irAEs of a lower grade were able to resume ICI therapy.

**Conclusion:**

Although the onset of irAEs was difficult to be predicted based on pre-treatment tests. It appeared that the continuation of ICI therapy, along with early detection and adequate control of irAEs, might contribute to the improved prognosis of patients.

## Background

The anti-programmed cell death protein 1 (PD-1) antibodies nivolumab and pembrolizumab are immune checkpoint inhibitors (ICIs) that activate the anti-tumour cytotoxic activity of T cells by inhibiting the binding of the PD-1 receptor and programmed cell death protein ligand 1 (PD-L1). They are currently used for the treatment of a wide range of cancers [1]. However, the overall response rate is low [2, 3], PD-1 and PD-L1 signaling disruption by ICIs regenerates T-cell-mediated anti tumor immunity, producing durable anticancer effects in a subset of patients.

Their associated adverse events are also unique and are termed as immune-related adverse events (irAEs), which are different from the events observed in patients treated with conventional anti-tumour agents. In some cases, irAEs are serious and can even result in death [4]. irAEs attracted attention soon after the approval of ICIs, and since then, several reports have been published [5–7]. Although symptoms such as type 1 diabetes mellitus and severe diarrhoea had attracted attention, recent reports have indicated that the onset of irAEs contributes to the prognosis of patients [8–13]. However, many of the reports describe studies on malignant melanoma and lung cancer, for which ICIs were used ahead of the use for other cancers. The sample sizes in the reports on malignant melanoma are small, and only small-scale studies have been reported on lung cancer. In the present study that involved a larger number of patients treated with anti-PD-1 antibodies for a wide range of cancers, we retrospectively investigated irAEs and therapeutic effects and determined whether the onset of irAEs could be predicted.

## Methods

### Study approval

The present study was reviewed and approved by the Ethics Committee of Fujita Health University (HM19–209). Informed consent was obtained from the eligible patients by an opt-out procedure. Using electronic medical records, we retrospectively evaluated patient characteristics (i.e. age, sex and Eastern Cooperative Oncology Group (ECOG) Performance Status (PS)), pre-treatment blood test data, presence or absence of irAE onset, the timing of irAE onset, the severity of irAEs, progression-free survival (PFS), overall survival (OS) and objective response to treatment.

### Patient characteristics

The patients were divided into two groups: the irAE group and the non-irAE group. Side effects with a high probability of having an underlying immunological basis, as documented by the treating provider and warranting frequent monitoring or potential intervention, were labeled as irAE.To determine the association of irAEs with the patient characteristics and pre-treatment test data, the survival times of the patients in these groups were compared. Moreover, the treatment and outcomes after the onset of irAEs, as well as differences among cancer types, were examined.

### Treatment and assessment

Nivolumab was administered via an intravenous infusion at a dose of 3 mg/kg every 2 weeks until August 2018 and after then, at a dose of 240 mg/bodyweight every 2 week according to recommended dosage changed. Pembrolizumab was administered at a dose of 200 mg/bodyweight every 3 weeks.Both drugs were administered until disease progression or the onset of uncontrollable adverse events. Adverse events were assessed according to the National Cancer Institute-Common Toxicity Criteria (NCI-CTC) version 4.03. Clinical response to treatment was categorized as either complete response (CR), partial response (PR), stable disease (SD), or progressive disease (PD) according to the Response Evaluation Criteria in Solid Tumors (RECIST) version 1.1. Although the timing of computed tomography analysis varied among cancer types, it was generally performed every 6–10 weeks. The baseline was adopted at the start point of ICI treatment. The measurable lesions were evaluated by the criteria of RECIST. Images of best response during the entire treatment period was used for this evaluation. Unsuitable for evaluation (e.g. loss of CT date or poorly timed examination) were clinically evaluated by corresponding clinician, we adopted the radiologist’s report and the doctor’s record for evaluation. Otherwise, the investigator made a discussion directly with the doctor to evaluate the clinical effect. The best overall response rates (ORR) were based on combining CR and PR disease response, DCR was based on combining CR,PR and SD disease response.

PFS was defined as the time from the start of immunotherapy to the date of disease progression or death, whichever occurred first. Patients who were alive without disease progression were censored on the date of last scan. OS was defined as the time from the start of immunotherapy to death Patients who were still alive were censored at the date of the last contact. Survival analysis was performed in November 2019, 8 months after the final enrollment of eligible patients.

### Statistical analysis

To compare the two groups, a t-test was performed for normally distributed data, and Mann–Whitney U and chi-square tests were performed for the other data. PFS and OS were compared using the Kaplan–Meier method and the log-rank test. The objective response rate (ORR) was defined as the best response after the initiation of the anti-PD-1 antibody therapy. The analysis software used was SPSS. In all analyses, a *P*-value of < 0.05 was considered to indicate a significant difference.

## Results

### Patient characteristics

Anti-PD-1 antibody therapy was administered to 280 patients with advanced cancer. The median age at the time of treatment was 67.5 (22–88) years, and the male-to-female ratio was 3:1. There were 59 patients with gastric cancer, 129 with lung cancer, 27 with renal cancer, 26 with head and neck cancer, 13 with malignant melanoma, two with Hodgkin’s lymphoma, four with malignant pleural mesothelioma, 13 with bladder cancer and seven with ureteral cancer. Nivolumab was used in 179 patients and pembrolizumab was used in 101. Nivolumab was administrated as the first line in 8 patients of malignant melanoma and 1 patient of renal cancer combined with nivolumab + ipilimumab. Pembrolizumab was given 27 patients of lung cancer as the first line treatment. All Hodgkin lymphoma patients were recurrent cases.

### Onset of irAEs

irAEs occurred in 115 patients (41.1%), and grade 3 and 4 irAEs occurred in eight patients (2.8%). The most common irAE was skin disorders (20.7%), many of which were a rash accompanied by pruritus. The second most common irAE was thyroid disorders (10.7%), which showed patterns of acute thyroiditis and hypothyroidism. Furthermore, interstitial pneumonitis was observed in 9.6% of the patients, many of which had lung cancer or head and neck cancer. Table 1 shows the patient characteristics according to patients with and without irAEs, and Table 2 summarises irAE symptoms. Based on consensus guidelines of American Society of Clinical Oncology [14], low grade irAE were discontinuation of ICI and follow-up, and grade 2 or higher adverse events used steroids. In addition, treatment was performed in multiple occupations by the intervention of specialists such as endocrinology and neurology. Only 1 patient used infliximab.

**Table 1:** Characteristics of patients with and without irAE

	with irAE(*n* = 115)	without irAE(*n* = 165)	*p* value
Age median (range)	69.7 (43–88)	66.0 (22–87)	0.2
Sex (male/female)	90/25	119/46	0.25
ECOG PS, 0/1/> 2	69/42/4	101/58/6	
preexisting autoimmune disease	10	14	0.26
past regimen line (%)			
1st	27 (23.5)	24 (14.5)	
2nd	43 (37.4)	47 (28.5)	
3rd	30 (26.1)	62 (37.6)	
4th^~^	15 (13.0)	32 (19.4)	
origin number (%)			
gastric	15 (13)	44 (26.7)	*p* = 0.03
lung	68 (59.1)	61 (36.9)	
RCC, urothelial and bladder cancer	19 (16.5)	28 (17)	
malignant melanoma	5 (4.3)	8 (4.8)	
head and neck	7 (6.1)	19 (11.5)	
reccurent Hodgikin lymphoma	1 (0.9)	1 (0.6)	
malignant pleural mesothelioma	0 (0)	4 (2.4)	
Best Response (number)			
CR	1	1	
PR	37	20	
SD	45	44	
PD	32	98	
ORR(%)	33	12.7	< 0.001
DCR(%)	72.2	38.2	< 0.001

Abbreviations: *CR* complete response, *PR* partial response, *SD* stable disease, *PD* progressive disease, *ORR* objective response rate, *DCR* disease control rate, *RCC* renal cell carcinoma

**Table 2:** iAE according to category and grade

Category	Total	%	G1–2	G3<
No, of irAE patients	115	41.1		
irAE cases (total)	145		137	8
Skin	58	20.7	56	2
rash	44	15.7		
itching	43	15.4		
vitiligo	3	1.1		
erythema	12	4.3		
herpes zoster	2	0.7		
Gastrointestinal	6	2.1	6	0
diarrhea	3	1.1		
stomatitis	3	1.1		
Thyroid	30	10.7	30	0
thyroiditis	12	4.3		
hypothyroidism	26	9.3		
Hepatobiliary	8	2.9	7	1
Renal dysfunction	4	1.4	4	0
Pneumonitis	27	9.6	24	3
Others				
pituitary	2	0.7	1	1
dabetes mellitus	3	1.1	3	0
myositis	1	0.4	1	0
ophthalmitis	1	0.4	1	1
encephalitis	1	0.4	1	0
hypoacusis	1	0.4	1	0
amnesia	1	0.4	1	0
neuropachy	2	0.7	2	0

### Therapeutic effects according to the onset of irAEs

**Fig. 1:**
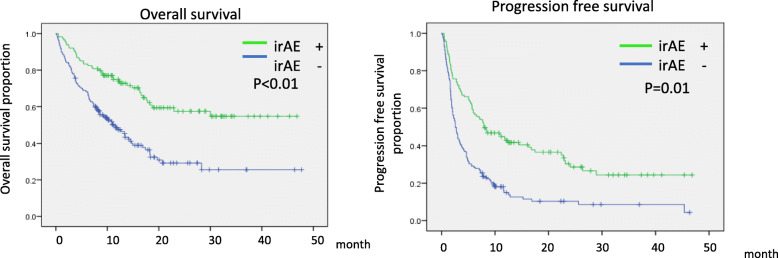
Kaplan-Meier survival curve of overall survival (OS) and progression free survival (PFS). OS and PFS following anti PD-1 antibody treatment in with irAE group(*N* = 115) and without irAE group (*N* = 165)

The median age of patients was 69.7 (43–88) in the irAE group and 66.0 (22–87) in the non-irAE group, and the patients in the irAE group were significantly older (Table 1, *P* = 0.024). The ORR was significantly higher in the irAE group (33.0%) than in the non-irAE group (12.7%; *P* < 0.001). The DCRwas higher in the irAE group (72.2%) than in the non-irAE group (38.2%; *P* < 0.001). The median OS was 48.7 months in the irAE group and 10.7 months in the non-irAE group (*P* < 0.01). The prognosis was significantly extended in the irAE group. The median PFS was significantly longer in the irAE group (17.6 months) than in the non-irAE group (3.0 months, *p* < 0.01; Fig. 1).

### Prediction of the onset of irAEs

Table 3 summarises the results of pre-treatment blood tests according to the presence or absence of irAEs. Although no significant difference was observed in the levels of rheumatoid factors with respect to the overall incidence of irAEs, the levels were higher in the irAE group (*P* = 0.08). In addition, the lactate dehydrogenase (LDH) levels were significantly lower in the irAE group (*P* = 0.02).

**Table 3:** Relationship between preexisting bood examination and irAE

	irAE(−)	irAE(+)	*p* value
KL-6			
n	139	87	0.7
U/mL	269 (182–458)	289 (196–424)	
ANA			
n	131	84	0.87
positive rate(%)	38.2	60.7	
TgAb			
n	124	75	0.22
IU/mL	0.9 (0.9–0.9)	0.9 (0.9–5.6)	
RF			
n	115	80	0.08
positive rate(%)	12.2	21.3	
anti AChR			
n	118	67	0.53
positive rate(%)	6.8	4.5	
TSH			
n	150	104	0.79
μU/mL	1.93 (0.95–3.09)	1.81 (1.08–3.28)	
FT3			
n	147	103	0.09
pg/mL	2.33 ± 0.51	2.44 ± 0.48	
FT4			
n	149	104	0.34
ng/dL	1.06 (0.96–1.22)	1.06 (0.94–1.18)	
ACTH			
n	114	66	0.95
pg/mL	27.7 (18.7–47.9)	30.8 (16.7–45.3)	
antiTPOA			
n	111	60	0.53
U/L	11.0 (9.0–12.0)	11.0 (9.0–13.0)	
AST			
n	162	115	1.49
U/L	21.0 (16.0–28.8)	21.0 (17.0–28.0)	
ALT			
n	163	115	0.51
U/L	14.0 (10.0–23.5)	15.0 (11.0–23.5)	
LDH			
n	160	109	0.02
U/L	213.5 (177.5–282.5)	198.0 (168.0–236.0)	
Alb			
n	156	111	0.2
g/dL	3.6 (3.1–3.9)	3.7 (3.2–4.0)	
CRP			
n	162	110	0.91
mg/dL	0.52 (0.11–2.27)	0.46 (0.17–1.86)	

Abbreviations: *ANA* antinuclear antibody, *RF* rheumatoid factor, *TgAb* thyroglobrin antibody, *Ach* Acetylcholine, *TPO* thyroid peroxydase

When irAEs were separately evaluated according to damaged organs, many patients with thyroid disorders had low levels of thyroid-stimulating hormones (TSHs) and free thyroxine (*P* = 0.02, both). Furthermore, the proportion of patients with high thyroglobulin antibody levels was higher in the irAE group (*P* = 0.05; Table 4).

**Table 4:** Relationship between irAE category and preexisting bood examination

	positive	negative	*p* value
Skin Toxicity			
anti Tg Ab	0.9 (0.9–11.0)	0.9 (0.9–0.9)	0.06
number	37	162	
antiTPOAb	11.0 (8.0–12.0)	11.0 (9.0–13.0)	0.31
n	31	142	
Thyroidtoxicity			
anti Tg Ab	0.9 (0.9–11.0)	0.9 (0.9–0.9)	0.05
n	24	175	
antiTPOAb	11.0 (8.8–13.3)	11.0 (9.0–12.5)	0.55
n	16	155	
TSH	2.40 (1.37–3.97)	1.51 (0.92–2.60)	0.02
n	33	200	
FreeT3	2.37 ± 0.54	2.37 ± 0.50	0.79
n	33	217	
FreeT4	0.98 (0.89–1.13)	1.06 (0.95–1.16)	0.02
n	33	200	
Pneumonitis			
KL6	274.5 (183.5–341.5)	283.0 (186.0–455.5)	0.568
n	22	200	

The incidence of irAEs was also compared among different cancer types. irAEs occurred in 65 patients with lung cancer (51.1%), and the incidence rate was significantly higher than in those with other cancer types (31.7%) (*P* < 0.001). In contrast, irAEs occurred in 15 patients with gastric cancer (25.4%), and the incidence rate was significantly lower than in patients with other cancer types (45.2%) (*P* < 0.01). When irAE symptoms were compared among cancer types, interstitial pneumonia occurred more frequently in patients with lung cancer, but no significant difference was observed between lung cancer and other cancer types. The incidence of liver disorders was higher in patients with gastric cancer than in those with lung cancer (*P* = 0.04). The observed grade 3 and higher adverse events were pneumonia, liver disorder, encephalitis and skin disorders. Although all patients with these adverse events had been hospitalised and treated with steroids, those with pneumonia and encephalitis subsequently died. Table 5 shows the outcomes of patients with high-grade adverse events. Of the 115 patients with grade 1 and 2 irAEs, 88 (76.5%) had discontinued ICI therapy and were treated with topical steroids and oral anti-histamines. ICI therapy was subsequently resumed and continued.

**Table 5:** Treatment outcome of severe irAE patients

organ	irAE detail	grade	origin	time to last ICI	Time to first ICI	treatment	outcome	reteatment	complication	preexisting antibody
skin	whole body prurigo	3	lung	21	69	steroid po&ointment	remission	try	none	RF
skin	whole body prurigo	3	lung	12	12	steroid po,ointment &iv	remission	stop	none	TgAb,TPO,RF, ANA
Liver	elevated liver enzyms	3	renal	21	21	steroid po	remission	PD	none	ANA
pneumonitis	interstitial pneumonia	4	lung	3	3	systemic pulse steroid	death	stop	dyalisis, lymphangitis	ANA
pneumonitis	interstitial pneumonia	4	gastric	16	140	systemic pulse steroid	death	PD	none	
pneumonitis	interstitial pneumonia	3	lung	7	31	systemic pulse steroid		PD	none	
CNS	convulsion	5	malignant melanoma	17	43	systemic pulse steroid	death	stop	brain meta RTx	
thyroid	hyposthenia	3	gastric	10	68	steroid po	remission	try	none	ANA

Abbreviations: *CNS* Central Nervous System

## Discussion

ICIs have been reported to be effective in treating various types of cancers. Although this provides great hope for patients, the response rates are low. Thus, studies are being conducted to identify biomarkers that can be used to predict therapeutic effects [15, 16]. Although PD-L1 is used clinically in patients with lung cancer, its effects have not been reproduced in other types of cancers. Recent studies have suggested that tumour mutation burden and microsatellite instability-high (MSI-H) can be used as biomarkers to predict the therapeutic effects. However, they are far from being established as biomarkers for some types of cancers. In recent years, several reports have indicated that the onset of irAEs is associated with the therapeutic effects in lung cancer, malignant melanoma and gastric cancer. However, these reports are based on small cohort studies with a dozen of patients because ICIs are infrequently used. In the present large-scale study involving 280 patients, we evaluated the association between irAEs and therapeutic effects, searched for predictive factors for the onset of irAEs and examined whether irAE profiles differ among cancer types. In this study, the overall incidence of treatment-associated adverse events was 41.1%, which was comparable with the previously reported incidence [5, 8, 9, 12]. The most common adverse events were skin disorders. In particular, a rash accompanied by dry skin and pruritus was frequently observed. However, grade 3 and higher skin disorders were observed in only two patients. In terms of severity, many patients with skin disorders could be treated with moisturising agents, oral anti-histamines and topical steroids. The skin disorders were not associated with eosinophil or lymphocyte counts. The second most common irAE was thyroid disorders. Thyroid disorders appeared in two patterns. In one pattern, the thyroid function was transiently enhanced by acute thyroiditis, followed by hypothyroidism. In the other pattern, hypothyroidism was detected in regular blood tests. Peiro et al. also reported similar findings [17]. Although Toi et al. [18, 19] and Maekura et al. [20] reported that thyroid peroxidase (TPO) antibody and thyroglobulin antibody (TgAb) levels before ICI therapy were predictive factors for the development of hypothyroidism, whereas our study showed no association with TPO antibody levels but suggested an association with TgAb levels (*P* = 0.05). Meanwhile, a high TSH level before treatment appeared to be a possible predictive factor for the development of hypothyroidism. Interstitial pneumonia occurred in approximately 7.3% of all patients and 3.4% of patients with gastric cancer, and its incidence tended to be even higher in patients with head and neck cancer and lung cancer. This higher incidence rate appeared to be associated with respiratory symptoms and ease of accidental ingestion. Hori et al. [21] also reported that skin disorders and pneumonia accounted for approximately 17.8% of adverse events in patients treated for head and neck cancer, which is consistent with our findings. The observed serious adverse events of grade 3 and higher were pneumonia, encephalitis, liver disorder and skin disorders. All patients with these serious adverse events were hospitalised and treated with steroids. Among them, two patients with pneumonia did not respond to pulse therapy and died. Regarding therapeutic effects, the ORR was significantly higher in the irAE group, and this was reproducible in a larger number of patients with either gastric or lung cancer. In addition, similar results were obtained for OS and PFS. Masuda et al. [12]., who examined patients with gastric cancer, reported that PFS was extended by ICI therapy, which is consistent with our study. Also Masuda et al. [12] reported in this paper, according to the waterfall plot showing tumour regression rates, all patients with a high rate had developed irAEs. Because significant differences were observed all in ORR, PFS and OS, pre-treatment tests themselves that allow the prediction of irAE onset may be possible biomarkers. In the present study, the comparison between patients with and without irAEs showed a significant difference only in LDH levels. By disorder, hypothyroidism was associated with high TSH and TgAb levels. This suggested that, in patients who already have hypothyroidism before ICI therapy, regardless of being symptomatic or asymptomatic, the probability of exacerbation after therapy could be estimated. Interstitial pneumonia was not associated with Krebs von den Lungen-6 (KL-6) levels at all. However, in some patients with pneumonia, KL-6 levels that were elevated after the onset of pneumonia were decreased following the treatment of pneumonitis, which suggested that KL-6 levels might be meaningful for the purpose of evaluating therapeutic effects. Identifying biomarkers for predicting the onset of irAEs is an issue that should be addressed in future studies [22]. Even among patients with irAEs, many of them resumed ICI therapy after treatment of these irAEs. It appeared that the continuation of ICI therapy, along with early detection and adequate control of irAEs, might contribute to the improved prognosis of patients.

The present study is a retrospective study, including patients with different treatment backgrounds; thus, it contains several confounding factors, which is a limitation. A larger scale prospective study, in addition to a search for biomarkers for predicting the onset of irAEs, should be conducted in the future.

## Conclusion

Although the onset of irAEs was difficult to be predicted based on pre-treatment tests. It appeared that the continuation of ICI therapy, along with early detection and adequate control of irAEs, might contribute to the improved prognosis of patients.

## Data Availability

The research data will not to be used for other purpose. The datasets generated during the current study are not publicly available due to ethical restrictions, but are available from the corresponding author on reasonable request.

## Abbreviations

ICI: Immune checkpoint inhibitor
PD-1: Programmed cell death protein-1
irAE: Immune-related adverse event
CTC-AE: Common Terminology Criteria for Adverse Events
PFS: Progression-free survival
OS: Overall survival
TSH: Thyroid-stimulating hormone
TPO: Thyroid peroxidase antibody
TgAb: Thyroglobulin antibody
ANA: Antinuclear antibody
RF: Rheumatoid factor

## References

[1] Kang YK, Boku N, Satoh T, Ryu MH, Chao Y, Kato K (2017). Nivolumab in patients with advanced gastric or gastro-oesophageal junction cancer refractory to, or intolerant of, at least two previous chemotherapy regimens (ONO-4538-12, ATTRACTION-2): a randomised, double-blind, placebo-controlled, phase 3 trial. Lancet.

[2] Gong J, Chehrazi-Raffle A, Reddi S, Salgia R (2018). Development of PD-1 and PD-L1 inhibitors as a form of cancer immunotherapy: a comprehensive review of registration trials and future considerations. J Immunother Cancer.

[3] Liu Y, Zhang T, Gao Y, Qu Y, Lu B, Zhang H (2019). A Single Center Analysis of Advanced Non-small Cell Lung Cancer Patients Treated with Immunotherapy in Real-world Practice. Zhongguo fei ai za zhi = Chinese journal of lung cancer.

[4] Myers G (2018). Immune-related adverse events of immune checkpoint inhibitors: a brief review. Curr Oncol.

[5] Zhou J, Wang H, Guo X, Wang Q, Duan L, Si X, et al. Management of immune checkpoint inhibitor-related rheumatic adverse events. Thoracic Cancer. 2019;11(1):198–202. 10.1111/1759-7714.13249 PMC6938753 31762209

[6] Spain L, Diem S, Larkin J (2016). Management of toxicities of immune checkpoint inhibitors. Cancer Treat Rev.

[7] Wang DY, Johnson DB, Davis EJ (2018). Toxicities associated with PD-1/PD-L1 blockade. Cancer J.

[8] Xing P, Zhang F, Wang G, Xu Y, Li C, Wang S (2019). Incidence rates of immune-related adverse events and their correlation with response in advanced solid tumours treated with NIVO or NIVO+IPI: a systematic review and meta-analysis. J Immunother Cancer.

[9] Aso M, Toi Y, Sugisaka J, Aiba T, Kawana S, Saito R, et al. Association between skin reaction and clinical benefit in patients treated with anti-programmed cell death 1 Monotherapy for advanced non-small cell lung Cancer. Oncologist. 2019;25(3):e536–e544. 10.1634/theoncologist.2019-0550 PMC7066688 32162801

[10] Das S, Johnson DB (2019). Immune-related adverse events and anti-tumor efficacy of immune checkpoint inhibitors. J Immunother Cancer.

[11] Lau KS, Liu R, Wong CC, Siu WKS, Yuen KK. Clinical outcome and toxicity for immunotherapy treatment in metastatic cancer patients. Ann Palliat Med. 2019;apm.2019.10.03. 10.21037/apm.2019.10.03 31735043

[12] Masuda K, Shoji H, Nagashima K, Yamamoto S, Ishikawa M, Imazeki H (2019). Correlation between immune-related adverse events and prognosis in patients with gastric cancer treated with nivolumab. BMC Cancer.

[13] Okada N, Kawazoe H, Takechi K, Matsudate Y, Utsunomiya R, Zamami Y (2019). Association between immune-related adverse events and clinical efficacy in patients with melanoma treated with Nivolumab: a multicenter retrospective study. Clin Ther.

[14] Brahmer JR, Lacchetti C, Schneider BJ (2018). Management of Immune-Related Adverse Events in Patiens treated with immune checkpoint inhibitor therapy: American Society of Clinical Oncology clinical guideline. JCO..

[15] Azuma Y, Nakaya A, Fujita S, Satake A, Nakanishi T, Tsubokura Y (2019). Neutrophil-to-lymphocyte ratio (NLR) fails to predict outcome of diffuse large B cell lymphoma. Leukemia Res Rep.

[16] Nakamura Y (2019). Biomarkers for immune checkpoint inhibitor-mediated tumor response and adverse events. Front Med.

[17] Peiro I, Palmero R, Iglesias P, Diez JJ, Simo-Servat A, Marin JA (2019). Thyroid dysfunction induced by nivolumab: searching for disease patterns and outcomes. Endocrine..

[18] Toi Y, Sugawara S, Kawashima Y, Aiba T, Kawana S, Saito R (2018). Association of Immune-Related Adverse Events with clinical benefit in patients with advanced non-small-cell lung Cancer treated with Nivolumab. Oncologist.

[19] Toi Y, Sugawara S, Sugisaka J, Ono H, Kawashima Y, Aiba T (2019). Profiling preexisting antibodies in patients treated with anti-PD-1 therapy for advanced non-small cell lung Cancer. JAMA Oncol.

[20] Maekura T, Naito M, Tahara M, Ikegami N, Kimura Y, Sonobe S (2017). Predictive factors of Nivolumab-induced hypothyroidism in patients with non-small cell lung Cancer. In vivo.

[21] Hori R, Shinohara S, Kojima T, Kagoshima H, Kitamura M, Tateya I (2019). Real-world outcomes and prognostic factors in patients receiving Nivolumab therapy for recurrent or metastatic head and neck carcinoma. Cancers..

[22] Patil PD, Burotto M, Velcheti V (2018). Biomarkers for immune-related toxicities of checkpoint inhibitors: current progress and the road ahead. Expert Rev Mol Diagn.

